# *Bombyx mori* silk/titania/gold hybrid materials for photocatalytic water splitting: combining renewable raw materials with clean fuels

**DOI:** 10.3762/bjnano.9.21

**Published:** 2018-01-17

**Authors:** Stefanie Krüger, Michael Schwarze, Otto Baumann, Christina Günter, Michael Bruns, Christian Kübel, Dorothée Vinga Szabó, Rafael Meinusch, Verónica de Zea Bermudez, Andreas Taubert

**Affiliations:** 1 Institute of Chemistry, University of Potsdam, D-14476 Potsdam, Germany; 2 Institute of Chemistry, Technical University Berlin, D-10623 Berlin, Germany; 3 Institute of Biochemistry and Biology, University of Potsdam, D-14476 Potsdam, Germany; 4 Institute of Earth and Environmental Science, University of Potsdam, D-14476 Potsdam, Germany; 5 Institute for Applied Materials (IAM) and Karlsruhe Nano Micro Facility (KNMF), Karlsruhe Institute of Technology (KIT), D-76344 Eggenstein-Leopoldshafen, Germany; 6 Institute of Nanotechnology (INT) and Karlsruhe Nano Micro Facility (KNMF), Karlsruhe Institute of Technology (KIT), D-76344 Eggenstein-Leopoldshafen, Germany; 7 Institute of Physical Chemistry, Justus-Liebig-University Giessen, D-35392 Giessen, Germany; 8 Department of Chemistry and CQ-VR, University of Trás-os-Montes e Alto Douro, Pt-5001-801 Vila Real, Portugal

**Keywords:** *Bombyx mori* silk, gold, photocatalytic water splitting, titania

## Abstract

The synthesis, structure, and photocatalytic water splitting performance of two new titania (TiO_2_)/gold(Au)/*Bombyx mori* silk hybrid materials are reported. All materials are monoliths with diameters of up to ca. 4.5 cm. The materials are macroscopically homogeneous and porous with surface areas between 170 and 210 m^2^/g. The diameter of the TiO_2_ nanoparticles (NPs) – mainly anatase with a minor fraction of brookite – and the Au NPs are on the order of 5 and 7–18 nm, respectively. Addition of poly(ethylene oxide) to the reaction mixture enables pore size tuning, thus providing access to different materials with different photocatalytic activities. Water splitting experiments using a sunlight simulator and a Xe lamp show that the new hybrid materials are effective water splitting catalysts and produce up to 30 mmol of hydrogen per 24 h. Overall the article demonstrates that the combination of a renewable and robust scaffold such as *B. mori* silk with a photoactive material provides a promising approach to new monolithic photocatalysts that can easily be recycled and show great potential for application in lightweight devices for green fuel production.

## Introduction

Fossil fuel availability is one of the pressing issues today. Especially in light of a growing world population and the corresponding increasing energy demand worldwide there is a need for alternative, sustainable, and cheap fuels [1–2]. Hydrogen (H_2_) is the most attractive fuel for fuel cells to produce “clean” electricity and water as an environmentally friendly reaction product [1,3]. However, one of the limitations of H_2_ is the efficient and sustainable H_2_ production. Currently, H_2_ is mainly produced by steam reforming of gas and oil, by catalytic reforming, or by water electrolysis [3–4].

In 1972 Fujishima and Honda reported that TiO_2_ is able to split water [5], a seminal discovery that has led to a wealth of studies on photocatalytic water splitting [6–10]. To be successful, the water splitting catalyst needs to have a certain set of properties. Most prominently, it should have a bandgap of at least 1.23 eV to provide the energy needed to split water. However, the bandgap should not exceed ca. 3 eV to most efficiently use the visible spectral range of the sunlight [6]. As a result, numerous water-splitting catalysts with various efficiencies have been reported [6–8,11–13].

Because of its bandgap of 3.0–3.2 eV (depending on the crystal structure and particle size [14–15]) TiO_2_-based water splitting catalysts are among the most popular materials for visible light water splitting [16–20]. Among these, TiO_2_/Au nanocomposites have attracted special interest because of their synergistic mode of action between the Au nanoparticle (AuNP) plasmons and the bandgap of the TiO_2_ semiconductors [21]. Gallo et al. used amorphous TiO_2_ doped with Au and/or platinum (Pt) NPs to split water under ultraviolet (UV)-A light and simulated sunlight. Best results with 1.6 mmol/(h·g) of H_2_ production were obtained with Au_0.5_Pt_0.5_/TiO_2_ catalysts [22]. Chen et al. used calcined P25 TiO_2_ NPs (TNPs) loaded with 3 wt % of Au for photocatalytic water splitting. Irradiation with UV and visible light combined yielded a higher H_2_ and oxygen production after 7 h than with UV or visible light alone [23].

In a more analytical study, Silva et al. [24] investigated the influence of particle size and preparation procedure on the photocatalytic activity of Au/TiO_2_ catalysts. Lower Au loadings of 0.25% Au on P25 TiO_2_ produced more efficient water splitting catalysts than materials with Au loadings of 1.5% or 2.2%. Moreover, the authors showed that 532 nm laser light is more efficient for water splitting than polychromatic light with wavelengths (λ) > 400 nm. Furthermore they also concluded that small Au particle sizes of around 2 nm and a low calcination temperature of 200 °C also yields materials producing more H_2_ than materials with larger Au particles or materials pre-treated at higher temperatures [24].

Following the same general idea, Gärtner et al. investigated the effect of different Au precursors for Au loading via in situ photodeposition with sodium tetrachloridoaurate(III) dihydrate (NaAuCl_4_·2H_2_O) providing the most efficient water splitting catalyst. The same authors also showed that methanol is the most efficient sacrificial agent when compared to isopropanol, glycerol, and glucose [25]. A further study by Jose et al. focused on the effect of TiO_2_ modification (anatase and/or rutile). These authors observed that TiO_2_ P25/Au mixed systems performed best in the entire UV–visible range when the TiO_2_ is composed of 75% of anatase and 25% of rutile with identical AuNPs [26].

In an interesting new approach, Zhang et al. found that Janus particles (rather than the core–shell or randomly organized materials described so far) based on large TiO_2_ particles with diameters of ca. 440 nm and AuNPs with diameters of ca. 60 nm could split water in the visible range, even at low Au fractions of only 1.8% [27]. Finally, Seh et al. showed that TiO_2_/Au Janus nanoparticles have a higher photocatalytic activity than the corresponding core–shell particles [28].

All materials described so far are powders or NPs. As a result, recycling is rather difficult and other photocatalysts that can more easily be recycled and are suited for continuous processes are necessary. For example, Liu et al. used a titanium sheet to make TiO_2_ nano-sheet films doped with different amounts of silver (Ag) NPs or TiO_2_ nano-grass films with AuNP for photocatalytic water splitting [29–30]. Matsuoka et al. sputtered TiO_2_ on a quartz glass plate by radiofrequency (RF) magnetron sputtering deposition followed by Pt deposition to make a TiO_2_ thin film photocatalyst for water splitting [31]. Finally, Goutailler et al. deposited an anatase/brookite NP mixture on cellulose from tetrabutylammonium bromide (N(*n*-Bu)_4_Br)/titanium tetraisopropoxide (Ti(OiPr)_4_) solutions in hexane [32]. These NPs strongly interact via non-covalent interactions (e.g., hydrogen bonds [33–34]) with the cellulose fibers and the resulting material was thus a macroscopic and mechanically robust object that could simply be retrieved and washed before reuse [32].

Another viable approach for the synthesis of larger and mechanically stable objects with photocatalytic activity is the immobilization of TiO_2_/Au nanostructures on a scaffold, ideally a scaffold with a high porosity, and good chemical and mechanical stability to enable access of all reactants to the catalytically active sites over extended periods of time. Moreover, a suitable photocatalyst should ideally use a scaffold from renewable materials; ideally the scaffold should be mechanically and chemically stable to withstand the conditions during photocatalysis.

Daoud and Xin [35] coated cotton fibers with 20 nm anatase particles via a sol–gel method. The covalently bonded TiO_2_ particles have a 50+ UV protection factor and are stable against washing. In an alternative approach, Zheng et al. used tetrabutylorthotitanate, Ti(OBu)_4_, and bis(*P*,*P*-bis-ethylhexyldiphosphato)ethanediolatotitanate (BPET, C_34_H_74_O_16_P_4_Ti) in a cold oxygen plasma to coat *B. mori* silk fabrics with TiO_2_ to produce a UV resistant material [36].

The current study also focuses on *B. mori* silk rather than cotton as a scaffold for photocatalyst synthesis. Instead of plasma chemistry, however, we employed a much softer wet chemistry method for materials synthesis.

*B. mori* silk contains numerous amino acids, predominantly glycine, alanine, serine, and tyrosine [37–38]. As numerous amide and hydroxyl groups are present in *B. mori* silk, a strong interaction of the silk scaffold with the TiO_2_ phase can be expected although silkworm silk does not contain specific TiO_2_ or Au binding domains [39–48]. Moreover, silk worms can easily be grown in large quantities and the resulting silk is chemically and mechanically rather robust [49]. As a result, the method reported here is a promising approach towards new photocatalytically active materials based on a renewable scaffold.

## Experimental

### Chemicals

*Bombyx mori* silk cocoons (http://www.seidentraum.biz, date of access: 14.01.2017), calcium chloride dihydrate (CaCl_2_·2H_2_O, ≥99%, Carl Roth), ethanol (EtOH; 99%, VWR), ethyl acetoacetate (EtAcAc, 99%, Alfa Aesar), glutaraldehyde solution (GA, 25% in H_2_O, Sigma-Aldrich), hydrogen tetrachloridoaurate(III) trihydrate (HAuCl_4_·3H_2_O, 99.99%, Alfa Aesar), poly(ethylene oxide) (PEO_780_, nominal *M*_w_ = 4600 g/mol, Sigma-Aldrich, measured *M*_w_ = 780 g/mol), PEO_8300_, (nominal *M*_w_ = 600 g/mol, abcr, measured *M*_w_ = 8300 g/mol), sodium carbonate (Na_2_CO_3_, 99.8%, Carl Roth), titanium isopropoxide (Ti(OiPr)_4_, TTIP, 98%, abcr), disodium hydrogen phosphate (Na_2_HPO_4_, ACS reagent, Sigma-Aldrich), magnesium sulfate (MgSO_4_, AnalaR Normapure, VWR), sodium phosphate monobasic (NaH_2_PO_4_, ACS reagent, Sigma Aldrich), uranyl acetate (p.a., Merck), ethanol absolute (EtOH_abs_, ≥99.8%, Carl Roth), acetone (≥99.8%, Carl Roth), AGAR Low Viscosity Resin (Plano), and PLANOCARBON (Plano) were used as received. Water-free solvents were stored over 3 Å molecular sieves prior to use. All syntheses were done with Millipore water (18.2 MΩ/cm).

### Synthesis

Materials synthesis is based on a significantly modified protocol by Hasegawa et al. [50–51]. The resulting materials are denoted as TS, TS_Au_x_, TPS, and TPS_Au_x_ (Table S1, Supporting Information File 1), where TS represents materials based only on TiO_2_ and silk, TPS stands for materials based on TiO_2_, PEO, and silk, TS_Au_x_ and TPS_Au_x_ denote the respective silk/TiO_2_ scaffolds further modified with Au and x denotes the amount of HAuCl_4_·3H_2_O (in mg) used in the synthesis.

**Step 1:** *Bombyx mori* silk cocoons were treated in 0.1 M aqueous Na_2_CO_3_ solution for 1 h at reflux temperature to remove the sericin. Subsequently, the cocoons were washed three times in hot water and dried at 40 °C in air.

**Step 2:** Silk was dissolved in a mixture of CaCl_2_/EtOH/H_2_O (molar ratio 1:2:8) at 60 °C (1 g silk/6.7 g solvent) [52] (solution 1) and 0.2 g of PEO_780_ and 0.2 g of PEO_8300_ were mixed and dissolved in 0.5 mL of water at 90 °C (solution 2). Then, 3.08 g of solution 1 and 2 mL of a 0.5 M CaCl_2_ solution were added to the hot solution 2. This mixture was briefly shaken at room temperature and then transferred to a 6 cm-diameter Teflon dish.

**Step 2a:** In an alternative reaction (to evaluate the effect of PEO), a PEO-free synthesis was also studied. In this case 6.16 g of the silk solution 1 and 2 mL of the 0.5 M CaCl_2_ solution were mixed and transferred to a Teflon dish as described before.

**Step 3:** 5 mL of Ti(OiPr)_4_, 4.6 mL of EtAcAc, and 1.4 mL of EtOH were mixed in a beaker. After 30 min 0.5 mL of a 0.5 M CaCl_2_ solution and 0.5 mL of water were added and the resulting slightly yellow solution was held in an oven at 60 °C for 9 min. This hot TiO_2_ precursor solution was immediately added to the (i) PEO/silk (step 2) or (ii) the PEO-free silk solution (step 2a) in the Teflon dish and stirred manually with a wooden spatula until a homogeneous liquid was obtained. The Teflon dish was covered with a glass Petri dish and left on the laboratory bench over night at room temperature.

**Step 4:** The resulting slightly yellow solid was purified via solvent exchange with EtOH/H_2_O (1:1 v/v), EtOH/H_2_O (3:7 v/v) each for at least 8 h at 60 °C, and finally with water for 24 h.

**Step 5:** When a modification with AuNP was desired, the as-prepared wet silk/TiO_2_ hybrid materials obtained in step 4 were stored in an aqueous HAuCl_4_ solution (10, 5, or 2.5 mg in 50 mL of water) overnight. No additional reducing agent was added to this reaction mixture. After 2 days the surface color changed from yellow to purple, indicating the deposition of AuNPs on the surface. The as-prepared Au/TiO_2_/silk hybrid material was washed three times with 50 mL of water at room temperature.

### Characterization

Samples for all transmission electron microscopy (TEM) investigations were prepared as follows: small pieces (ca. 2 mm^2^) of the wet hybrid materials were immersed in a 3% glutaraldehyde solution in 0.1 M phosphate buffer (pH 7) for 30 min followed by washing three times in 0.1 M phosphate buffer, once with water, and once with 50% ethanol for 5 min each. Then the hybrid materials were immersed in 2% uranyl acetate solution in 50% ethanol for 30 min followed by treatment with 70% EtOH, 90% EtOH, 100% EtOH, 2× 100% EtOH_abs._, and 2× 100% acetone_abs._ for 15 min each. Finally, the materials were immersed in Agar Low Viscosity Resin/acetone (1:2 wt/wt) mixtures for 2 h, then in resin/acetone (1:1 wt/wt) mixtures for 2 h, and then in pure resin overnight under a rotatory motion. The as-prepared samples were transferred into a mold, covered with resin, and treated at 60 °C for 24 h. After cooling to room temperature, the embedded samples were trimmed and sectioned (100 nm nominal slice thickness, Leica Ultracut UCT with diamond knife at room temperature).

Transmission electron microscopy (TEM) was done on a Philips CM 200 TEM with a LaB_6_ cathode operated at 120 kV. High resolution transmission electron microscopy (HRTEM) was done using an aberration corrected Titan 80-300 (FEI, Eindhoven, The Netherlands) with field emission gun, operated at 300 kV. Scanning transmission electron microscopy (STEM) and chemical analysis were done on a Tecnai F20 ST (FEI, Eindhoven, The Netherlands) field emission TEM equipped with an Orius SC600 CCD-camera and a S-UTW EDX detector (EDAX, Mahwah, NJ, USA) operated at 200 kV. For EDXS the instrument was in scanning transmission mode and the α-tilt of the samples was set to 20°.

Scanning electron microscopy (SEM) and energy dispersive X-ray spectroscopy (EDXS) were performed on a JEOL JSM-6510 with a W filament operated at 15 kV and equipped with an Oxford Instruments INCAx-act detector. Dry samples were either ground and deposited as powders on a carbon glue pad followed by sputtering with carbon using a Polaron CC7650 Carbon Coater, or, alternatively, deposited directly on the carbon glue pad using PLANOCARBON and sputtered with Pd/Au using a SC7620 Mini Sputter Coater.

X-ray photoelectron spectroscopy (XPS) measurements were done on a K-Alpha+ XPS instrument (Thermo Fisher Scientific, East Grinstead, UK). Data acquisition and processing using the Thermo Avantage software is described elsewhere [53]. All samples were analyzed using a micro-focused, monochromated Al Kα X-ray source (30–400 µm spot size). The K-Alpha+ charge compensation system was employed during analysis, using 8 eV electrons and low-energy argon ions to prevent any localized charge build-up. The spectra were fitted with one or more Voigt profiles (binding energy uncertainty: ±0.2 eV). The analyzer transmission function, Scofield sensitivity factors [54], and effective attenuation lengths (EALs) for photoelectrons were applied for quantification. EALs were calculated using the standard TPP-2M formalism [55]. All spectra were referenced to the C1s peak of hydrocarbons at 285.0 eV binding energy controlled by means of the well-known photoelectron peaks of metallic Cu, Ag, and Au. Sputter depth profiles were obtained using a raster scanned Ar^+^ ion beam at 0.5–3.0 keV and 30° angle of incidence.

X-ray powder diffraction (XRD) was performed on a PANalytical Empyrean Diffractometer in a 2θ range of 4–90°. X-ray wavelength was 1.5408 Å (Cu Kα) and step size was 0.0131°. Data and particle size analysis via Scherrer equation was done using the HighScore Plus V.4.0 (4.0.0.19037) software from PANalytical B.V.

Nitrogen sorption measurements were performed on a BELSORP-max with N_2_ at 77 K using 40 measurement points. Prior to all measurements, samples were ground and dried at 90 °C for at least 24 h until the weight differences between two subsequent measurements were less than 10%. Data analysis was done with the BELMaster™ 6.3.0.0 software from BEL Japan, Inc.

Mercury (Hg) intrusion porosimetry was done on a Pascal 140/440 porosimeter (Thermo Fisher Scientific, Rodano, Italy) in a pressure range of 0–400 MPa. The instrument software (Sol.I.D.) was employed for calculations of results supporting Washburn’s equation. A mercury surface tension of 0.48 N/m and a contact angle of intruded mercury of 140° were assumed.

Reflection solid-state UV–vis spectrometry was done on a Perkin-Elmer (PE) Lambda 950 UV–vis spectrometer from 200–850 nm with a resolution of 2 nm using a Praying Mantis™ attachment from Harrick Scientific Products Inc. Ground samples were mixed with MgSO_4_ in a 1:10 w/w ratio prior to measurements. Data analysis was done with the PE UV WinLab V6.03 software.

Fourier transform attenuated total reflection infrared spectroscopy (FT-ATR-IR) was done from 4000–500 cm^−1^ with a resolution of 2 cm^−1^ on an FT-IR NEXUS spectrometer with a ThermoNicolet SmartOrbit ATR attachment with a diamond crystal. Samples were directly deposited on the crystal und fixed in the measurement position via the SmartOrbit attachment. Data analysis was done with the ThermoFisher Omnic 8.1.11 software.

Raman spectroscopy was performed on a WITec alpha300 confocal Raman microscope with an upright optical microscope. Laser wavelength was 532 nm and laser power was 14 mW. The laser was coupled into a single mode optical fiber and focusing on the sample was achieved via an Olympus MPlanFL N (NA = 0.9) 100× objective yielding a probe area of 1.3 µm^2^ [56]. Raman spectra were obtained with an integration time of 50 s from 0–1200 cm^−1^ and the grating of the spectrograph was set to 1800 g/nm to avoid laser-induced damage in the samples. Data analysis was done with the WITec Control FOUR 4.1 software.

Thermogravimetric analysis (TGA) was done on a Netzsch TG 209F1 from 30 °C to 1000 °C with a heating rate of 10 K/min in synthetic air. To evaluate the reproducibility of the measurements, one sample was analyzed three times. All data showed the same behavior and the deviations between the individual measurements were below 1%. Elemental analysis (EA) for C, H, and N was performed on a Vario EL III (elementar).

Photocatalysis experiments were done in a planar photoreactor with defined geometry consisting of a stainless steel housing with cooling, a Teflon inlet as reaction chamber, and a 6 cm diameter quartz glass window [57]. The irradiation area is given by the size of the solid sample that was irradiated. A solar simulator (LOT Oriel Quantum Design), which is basically a 300 W Xe-lamp equipped with an AM-1.5G filter, was used at an intensity of about 1000 W/m^2^ as light source. For comparison a 300 W Xe-lamp without the AM1.5G filter was also used.

In a typical experiment, the catalyst and a stirring bar were placed in the reactor. Then the setup was evacuated three times and filled with argon (Ar) to remove residual oxygen. Thereafter, 53 mL of a 2:1 mixture of water and ethanol were added under an Ar stream, and the thermostat was set to 25 °C. Both water and ethanol were treated with Ar for at least 10 min to remove dissolved oxygen before injection into to the reaction chamber. The light source was placed at a distance of 10 cm. After reaction (usually 24 h), a sample of the gas phase was analyzed in a gas chromatograph equipped with a Carboxen column (Agilent Technologies 7890A GC System with a jasUNIS Injector System) to determine the amount of H_2_.

## Results

Two types of materials were made. The first group only contains TiO_2_ (T) and silk (S), and will be denoted “TS” throughout the remainder of text. The second group contains TiO_2_ (T), PEO (P), and silk (S), and will be denoted “TPS”. The respective gold-modified materials are denoted TS_Au_x_ and TPS_Au_x_, respectively, where x is the amount of HAuCl_4_·3H_2_O used for the synthesis in milligrams. Table S1, Supporting Information File 1 summarizes the materials studied in this work.

**Figure 1:**
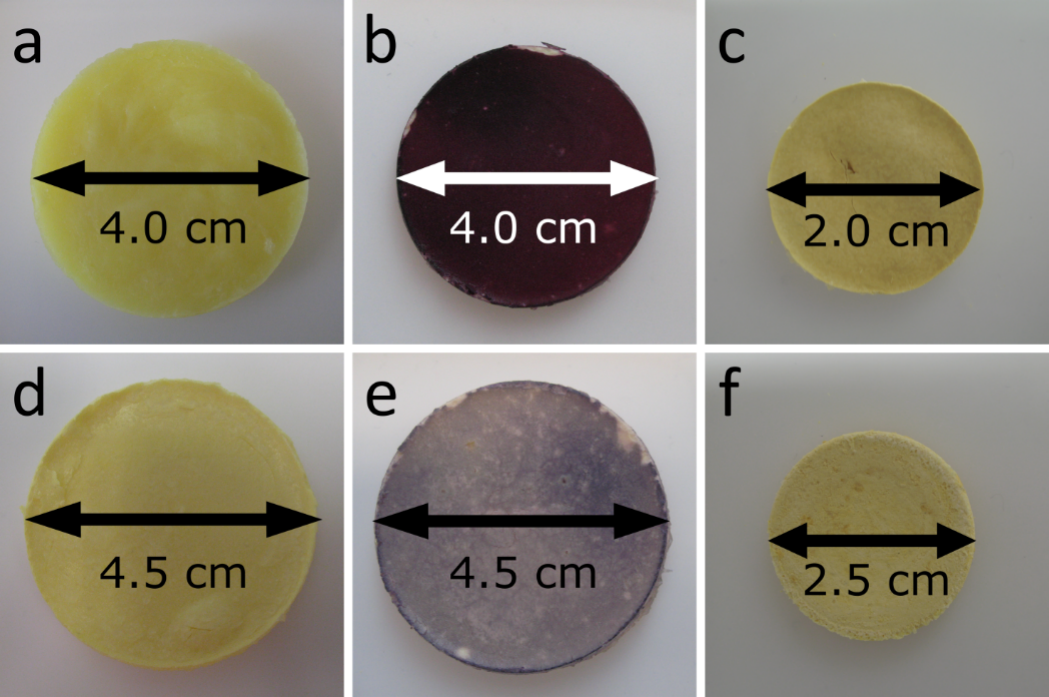
Hybrid materials: a) wet TPS, b) wet TPS_Au_2.5_ c) dry TPS, d) wet TS e) wet TS_Au_2.5_ f) dry TS.

Figure 1 shows photographs of the hybrid materials. The wet TS and TPS materials are slightly yellow and have a diameter of 4.0–4.5 cm (the Teflon dishes used for synthesis have a diameter of 6 cm). After drying, the samples lose 70–75 % in weight and shrink to a diameter of 2.0–2.5 cm. All samples remain intact and appear macroscopically homogeneous.

After Au deposition, the surface color of the TPS samples changes to purple. The color depth relates to the amount of HAuCl_4_·3H_2_O used for the synthesis. The surface of TPS_Au_1.0_ is light purple; in the case of TPS_Au_2.5_ it is intense purple (Figure 1). The surface of TPS_Au_5.0_ is dark purple to nearly black, and TPS_Au_10.7_ has a surface with an Au cast. Moreover, light microscopy of cross-sections of the materials (Figure S1, Supporting Information File 1) suggests that the penetration depth of the AuNP reaches up to 120 µm, judging from the purple color visible in the optical micrographs.

The absence of PEO affects the color of the TS samples. TS_Au_2.5_ is light purple but the color is less intense than in TPS_Au_2.5_. Again, the variation of the Au salt concentration leads to a color change. In contrast to TPS_Au_x_, however, the surfaces of TS_Au_5.6_ and TS_Au_6.1_ do not show intensification of the purple color, but instead an increasing gray hue. Moreover, the penetration depth of the Au – again judging by the color – is 400 µm; this is larger that observed for TPS_Au_2.5_.

Finally, Au deposition does not influence the shrinking: both the TiO_2_/silk and the TiO_2_/silk/Au hybrids have roughly the same diameters in the wet and the dry states, respectively. Moreover, no color change is observed on drying.

**Figure 2:**
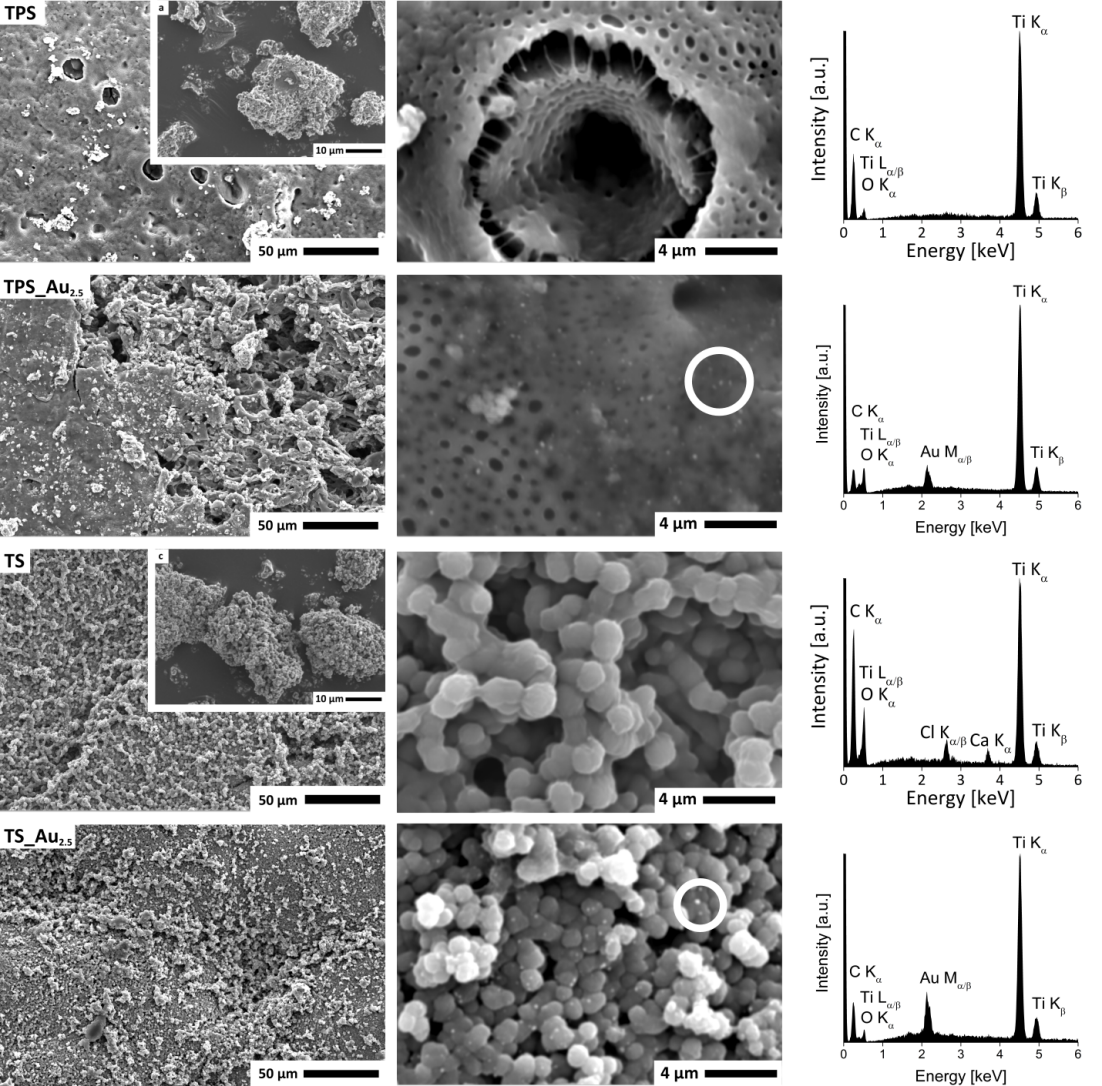
From top to bottom: SEM Images (left) of the surfaces of TPS, TPS_Au_2.5_, TS, and TS_Au_2.5_ at different magnifications, and EDX spectra (right) of the respective samples. Insets show examples of the same material after grinding. Circles highlight some of the bright spots mentioned in the text.

Figure 2 shows scanning electron microscopy (SEM) and energy dispersive X-ray spectroscopy (EDXS) data obtained from the hybrid materials. TPS has a rough and rather dense surface with large holes up to 16 µm in diameter and some smaller aggregates on the surface. Higher magnification images show smaller holes with diameters ranging between 0.04 and 0.1 µm. These holes are often located around the larger holes described above. EDXS confirms the presence of Ti and O, suggesting the formation of TiO_2_. Finally, the samples also exhibit fiber-like features, presumably from the silk.

Au deposition appears to have promoted the opening of the surface of the samples to some extent. As a result, TPS_Au_2.5_ partially exhibits a macroporous structure and an inhomogeneous surface morphology with smaller particles located on the surface, similar to the Au-free sample TPS. Again smaller holes are visible around the larger holes. Moreover, the SEM images of TPS_Au_2.5_ show small spots (highlighted with white circles) that were not observed before Au deposition. These objects are assigned to AuNPs and EDXS indeed confirms the presence of Au in these samples.

The PEO-free hybrid materials (TS) have a different morphology. At lower magnification the sample surface has a grainy appearance but higher magnification images show that the materials consist of tightly connected spherical nanoparticles with significant open volume. The particles have a diameter of ca. 1.5 µm and appear to be composed of smaller particles.

The surface morphology of the gold-containing TS_Au_2.5_ is very similar to the morphology of the TS materials. Unlike the TS materials, however, all TS_Au_2.5_ materials show small, bright dots (highlighted with white circles) in the SEM images. These can again be assigned to AuNP. The presence of Au is again confirmed by EDXS.

EDXS generally only shows C Kα, O Kα, Ti Kα/β, and Ti Lα/β signals. Only the TS samples exhibit Ca Kα and Cl Kα/β signals showing that these samples also contain Ca and Cl. As both these elements are present in the reaction mixture (see Experimental section) it is likely that they are not completely washed out during purification.

**Figure 3:**
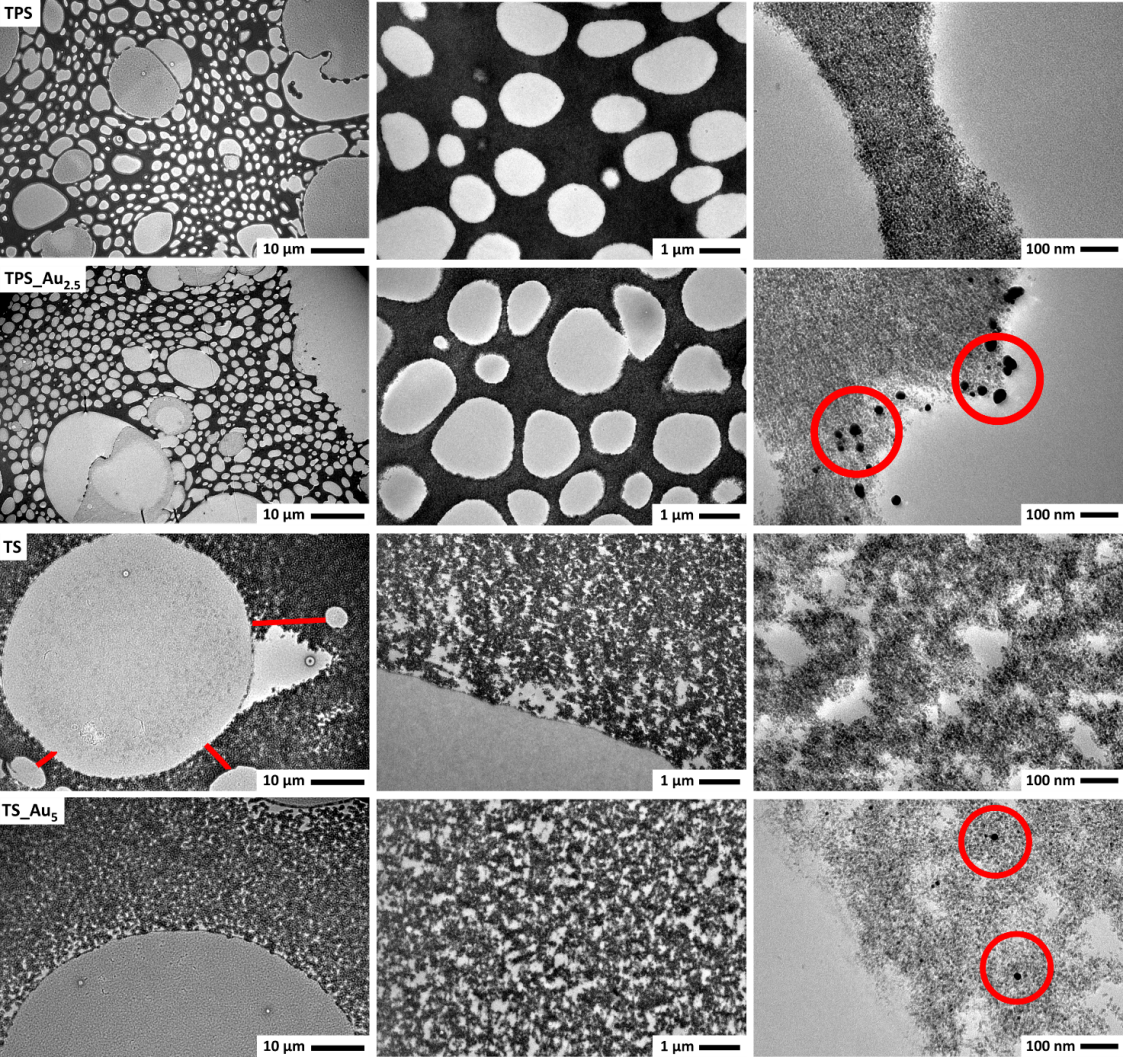
TEM images of TPS, TPS_Au_2.5_, TS, and TS_Au_5.0_ at different magnifications. Red circles highlight the AuNPs and the red bars highlight the walls between two adjacent pores in TS.

Figure 3 shows representative TEM images of the same materials. Lower magnification images reveal significant differences between the TS and TPS materials. The TPS materials exhibit tightly packed pores with diameters ranging between 0.4 and 4.0 µm and rather thin walls between the pores of ca. 0.1–1.8 µm. A few larger pores with diameters up to 50 µm are present as well (Figure S2, Supporting Information File 1). Some of these larger pores have a darker hue, suggesting that they are filled with silk, because the silk was stained with uranyl acetate. The lighter pores are likely filled with resin. The location of the PEO could not be determined.

The TS materials exhibit much less pores than the TPS materials. The pore diameters are larger (10 to 100 µm) than in the TPS materials. The pores are separated from one another by walls with thicknesses between 2–20 µm (highlighted by red lines in Figure 3). All pores show a light gray structure in the TEM images, indicating that they may be filled with silk.

Higher magnification images reveal that the inorganic structure surrounding these large pores is composed of TiO_2_ NPs (TNPs) with diameters on the order of 5.5 ± 1.5 nm (Figure 3). Although the primary particles roughly have the same size in all samples, there are differences in the inter-particle distances. While the NPs in the TS samples show a more open, cluster-like structure with small pore-like spaces up to 100 nm, the TPS samples always exhibit very densely packed NPs without further pores in the 10–100 nm range. In spite of their different arrangements, the individual TNPs are essentially identical in all the samples.

Unlike the TNPs, the AuNPs found in the TS and TPS materials are different. For example, in TPS_Au_2.5_ the nanoparticles are located at the edge and the pore walls of the hybrid material and are positioned fairly close to each other. The particles have a rather broad size distribution between 4–38 nm, with most particles in the 10–18 nm range (Figure 3).

Judging from the TEM images, TS_Au_5.0_ contains a lower number of AuNPs in spite of the fact that the amount of HAuCl_4_·3H_2_O used for the synthesis was higher than in TPS_Au_2.5_. Furthermore, the particles are more clearly separated from one another. TEM also suggests that the AuNPs have a higher penetration depth in the TS-based materials than in the TPS_Au_x_ materials, which is consistent with optical microscopy. Moreover, the diameter of the AuNPs in the TS_Au_5.0_ materials is mainly between 7–13 nm, but smaller Au particles are also present. Furthermore, bright field TEM images of TS_Au_5.0_ show a homogeneous AuNP distribution on the TNP.

Dark field STEM images of the HM show a better contrast between Au and TiO_2_ (Figure S3, Supporting Information File 1). The AuNP in TS_Au_5.0_ and TPS_Au_2.5_ are rather homogeneously distributed. Moreover, the images show that the size distribution of the AuNP is quite homogeneous and no overly large or highly aggregated particles are present in the samples. The slight differences between the AuNP distribution in TEM and STEM may be due to variation between sample areas.

Both the AuNPs and the TNPs were further analyzed via HRTEM and fast Fourier transformation (FFT) analysis of the observed lattice fringes along with further EDXS experiments. Figure S4, Supporting Information File 1 shows a representative HRTEM image of a typical AuNP and the surrounding TNP in TS_Au_5.0_. The size of the AuNP (dark spot) is 12 nm. The AuNP is surrounded by different TNPs of about 5 nm in diameter. The size was deduced from the extension of the lattice fringes observed in the HRTEM images (white circles). FFT analysis of the HRTEM image shows a series of reflections that can be assigned to Au and different TiO_2_ modifications (brookite and anatase), which are also identified by XRD (Table S2, Supporting Information File 1). Rutile cannot be detected. The EDX spectra were primarily obtained by spot analysis with the electron beam directly placed on one of the dark spherical features; the corresponding spectra show intense Au signals, which confirm HRTEM.

Corresponding data for TPS_Au_2.5_ are displayed in Figure S4, Supporting Information File 1. The AuNP shown here is 15 nm in diameter and the lattice spacings observed in this particle (0.235 nm and 0.204 nm) can again be assigned to (111) and (200) fringes of gold. The TNP observed in these samples have a size of roughly 5 nm in diameter and the lattice spacings obtained from FFT indicates that anatase and brookite are present in the sample. The corresponding EDX spectra prove the presence of Au and Ti.

**Figure 4:**
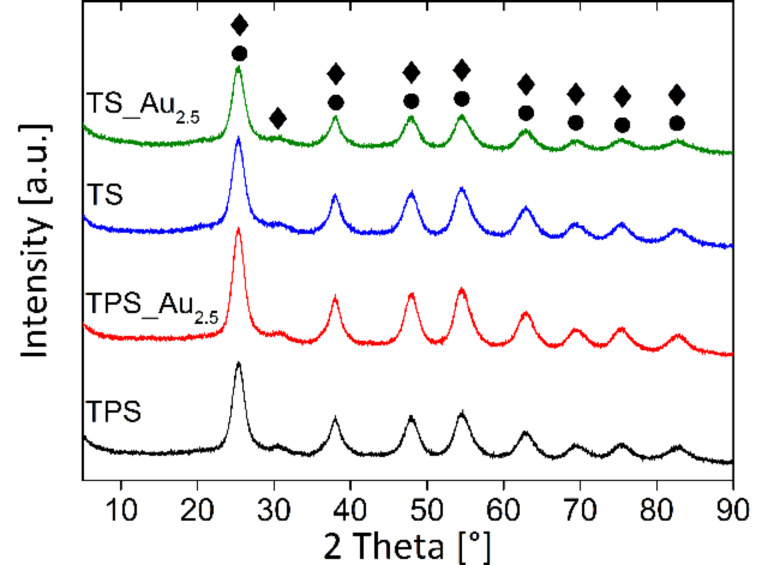
XRD patterns of the hybrid materials. Reflections labeled • are anatase reflections (ICDD: 98-015-4602) and reflections labeled ♦ are brookite reflections (ICDD: 00-029-1360).

Figure 4 shows representative XRD patterns of the hybrid materials. All patterns are essentially identical and exhibit a series of broad reflections that can be assigned to anatase (ICDD 98-015-4602). Only one reflection at 30.5°2θ is specifically due to brookite (i.e., the (121) reflection with 90% intensity, ICDD 00-029-1360). The other brookite reflections are located at the same positions as the anatase reflections and can therefore not be distinguished. The very low intensity of the brookite (121) reflection, however, suggests the presence of only a low amount of brookite.

Interestingly, the XRD patterns do not display any reflections assigned to Au. In principle, the most intense Au reflection ((111), ICDD 00-004-0784) should be observed at 38.2°. Unfortunately, this position overlaps with the anatase (004) reflection. Therefore, an unambiguous assignment of the observed reflection is not possible. Measurements for longer times and higher count rates for improvement of the signal to noise ratio were thus done from 35–47° (Figure S5a, Supporting Information File 1). The Au (200) reflection at 44.4° (ICDD 00-004-0784) should be clearly visible if high amounts of Au are present in the samples. There is, however, no evidence of this Au reflection. The weak reflection at 42.3° can be attributed to the brookite (221) reflection. Consequently XRD agrees with HRTEM which pointed out the presence of anatase and some brookite.

Scherrer analysis [58] of the XRD patterns yields an average anatase particle size of 4.0 ± 0.5 nm in all samples. This is in good agreement with particle sizes of 5–10 nm evident from the TEM images (Figure 3, Figure S3, Supporting Information File 1).

For photocatalytic water splitting (see below) the wet samples were used. To ensure that there is no drying-induced phase transition in the hybrid materials, XRD was also done on a wet sample (Figure S5b, Supporting Information File 1). Clearly, the wet samples produce a higher X-ray background due to large amounts of water present in these samples. Nevertheless, the data prove that there is no structural change in the material upon drying because XRD patterns are identical to the data obtained from the dry material. Moreover, particle size analysis via Scherrer equation [58] yields exactly the same particle size of 4.1 ± 0.5 nm for the wet samples.

**Figure 5:**
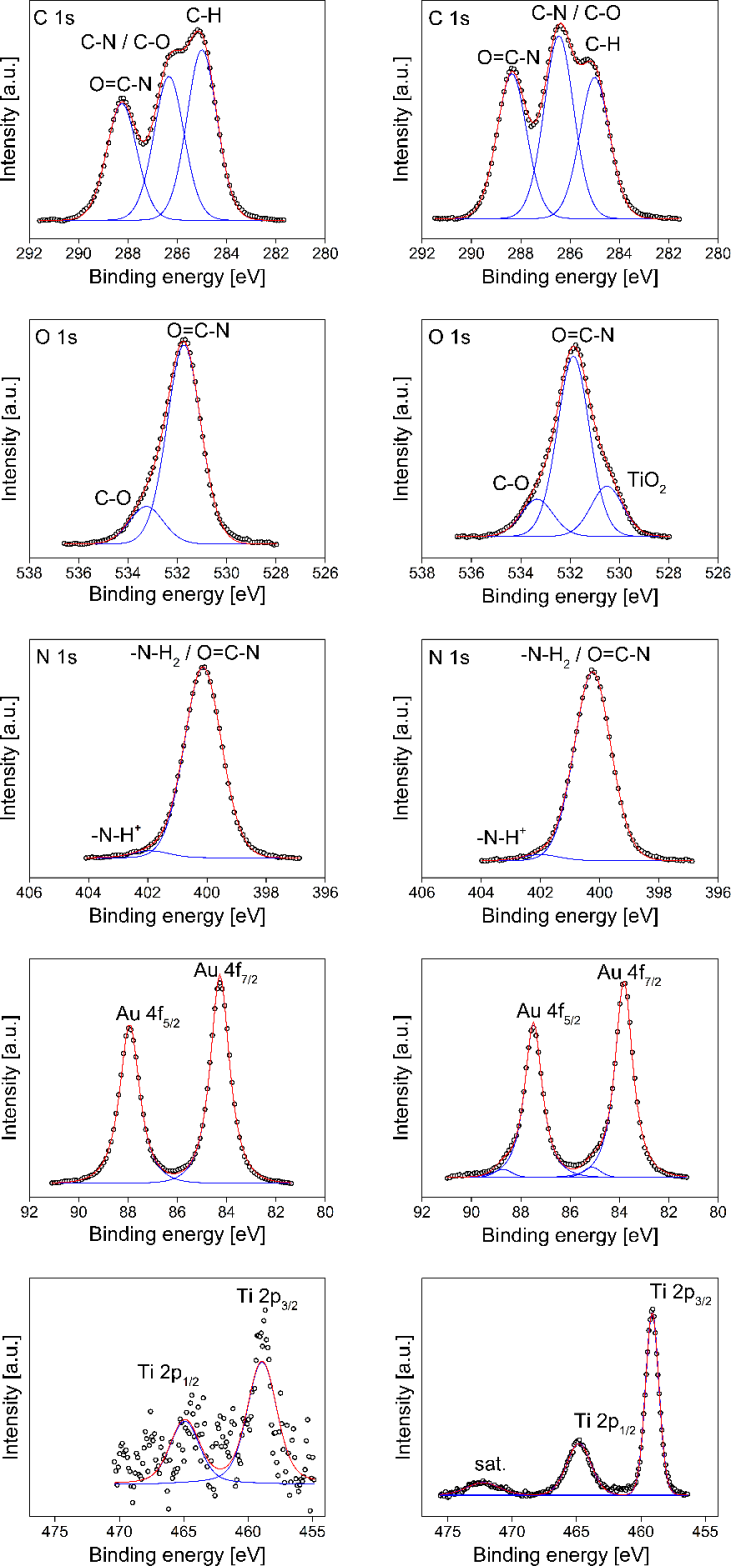
Au 4f, Ti 2p, C 1s, N 1s, and O 1s XP spectra of the TS_Au_2.5_ (left) and TPS_Au_2.5_ (right) surfaces. Open circles are experimental data, red line represents the sum of all fits and blue lines are the single fit components.

Figure 5 compares the Au 4f, Ti 2p, C 1s, N 1s, and O 1s XP spectra of the TS_Au_2.5_ and TPS_Au_2.5_ surfaces. The respective binding energy assignments (Table S3, Supporting Information File 1) are in a good agreement with literature. The main peak at Au 4f_7/2_ = 84.0 ± 0.2 eV is attributed to metallic Au, which proves the formation of Au^0^ NPs [59]. The additional weak component at Au 4f_7/2_ = 85.1 eV in case of TPS_Au_2.5_ cannot be attributed unambiguously, but is probably due to a local charge-up. The binding energy of Au^3+^ residues would be expected at Au 4f_7/2_ = 86.6 eV and, therefore, the presence of Au^3+^ species can be excluded [60].

The Ti 2p_3/2_ peak at 459.0 eV and the corresponding O 1s peak at 530.5 eV are attributed to TiO_2_ [61]. Furthermore the binding energy of Ti 2p_3/2_ at 459.0 eV could possibly also be assigned to Ti–O–C units, possibly stemming from silk, PEO, or residual EtAcAc connected to the TNPs [62]. The O 1s binding energies at 531.8 eV and 533.3 eV can be attributed to Ti–OH motives [63].

The main N 1s peak at 400.1 eV is attributed to the amide and amine groups (peptide bonds) of silk (Table S3, Supporting Information File 1) [64–67]. This assignment is corroborated by the corresponding C 1s peaks at 286.4 eV (C–N) and 288.3 eV (O=C–N, O 1s = 531.8 eV) [66–70]. The weak N 1s component at 402.0 eV is assigned to protonated nitrogen [68]. Note that C–N and C–O contributions cannot be resolved within the C 1s multiplet. In spite of this, the weak O 1s peak at 533.3 eV suggests the presence of C–O groups originating from PEO or synthesis residuals of EtAcAc [66,68]. The C 1s peak at 285.0 eV is attributed to the C–H groups of PEO and silk and adventitious carbon.

Table S3, Supporting Information File 1 summarizes the XPS binding energies, assignments to the respective binding partners, and atomic concentrations for the samples TS_Au_2.5_ and TPS_Au_2.5_. The chemical composition of all materials is fairly similar except for the fractions of Au and Ti. TS_Au_2.5_ has a higher amount of Au (2.2 atom % vs 1.2 atom %) whereas the amount of titanium (and hence TiO_2_) is lower (0.2 vs 2.1 atom %). Conversely, the Au content is lower in TPS_Au_2.5_ and the TiO_2_ content is higher.

Furthermore, XPS sputter depth profiles were acquired to obtain information on the chemical composition of the samples vs depth. Figure S6, Supporting Information File 1 shows a representative data set from TPS_Au_2.5_. The fractions of O and Ti increase slightly with increasing sample depth until a plateau is reached at around 500 s. The atomic concentrations of around 30% for Ti and around 60% for O indicate the presence of TiO_2_. In contrast the atomic concentrations of C, N, and Au are much lower and further decrease with increasing sample depth, indicating that Au and possibly silk are slightly enriched directly on the surface [71].

EDXS and XPS data (Figure 2 and Figure S4, Supporting Information File 1) were further confirmed by elemental analysis (EA, Table S4, Supporting Information File 1). The overall amount of CHN (indicative of the organic fraction in the materials) is 15.2% in TPS and 14.6% in TPS_Au_2.5_. The fraction of organic material is 21.2% in TS and 20.8% in TS_Au_2.5_.

**Figure 6:**
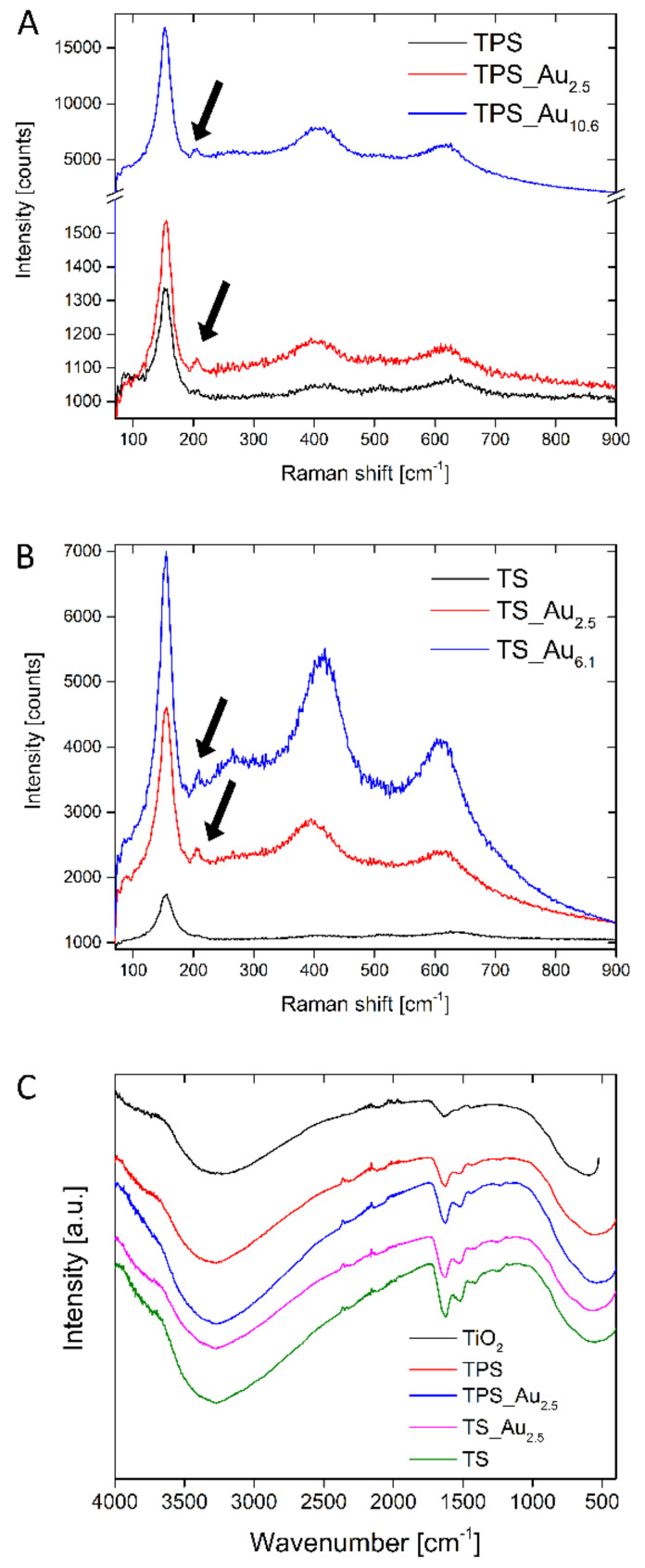
Raman spectra of (A) TPS_Au_x_ and (B) TS_Au_x_. (C) IR spectra of TiO_2_ made using the same procedure employed for the hybrid materials (black line), TPS (red), TPS_Au_2.5_ (blue line), TS_Au_2.5_ (violet line), and TS (green line). The signal around 2200 cm^−1^ in the IR spectra stems from the diamond crystal of the ATR-IR spectrometer.

Figure 6 shows complementary infrared (IR) and Raman spectra. The presence of AuNPs is advantageous, as the signals of chemical species close to the AuNPs are enhanced (surface enhanced Raman effect) [72–73] providing chemical information on the surroundings of the AuNP. In all cases, the addition of Au leads to an increase in the Raman band intensities compared to the Au-free samples. As a result, the Raman spectra of TPS_Au_x_ and TS_Au_x_ can be analyzed in detail.

The Raman spectra obtained for “bulk” anatase typically show O–Ti–O vibration bands at 144, 197, and 640 cm^−1^ from the E_g_ symmetric stretching vibration, at 400 and 519 cm^−1^ from the B_1g_, symmetric bending vibration, and at 513 cm^−1^ from the A_1g_ asymmetric bending vibration [63,74]. The bands at 519 and 513 cm^−1^ overlap in the current spectra although they are known to be separated below 73 K [63,74]. In the case of nanoparticles (vs bulk TiO_2_), broader signals and a red or blue shift in the Raman bands are observed [75]. The O–Ti–O vibration bands observed in Figure 6 are located at 626, 509, 409, 209, and 151 cm^−1^. The band at 151 cm^−1^ is the most intense and sharpest signal. The 209 cm^−1^ band is very weak and its intensity increases in the presence of Au. The bands at 409 and 630 cm^−1^ are broad and again their intensity increases in the presence of AuNP. In contrast, the band at 509 cm^−1^ is also broad, but there is no significant change in intensity upon Au addition. Furthermore, the Raman spectra of TPS_Au_10.6_ and TS_Au_6.1_ show a new weak, broad band at 266 cm^−1^ possibly originating from brookite (RRUFF ID: R50363.3), consistent with XRD.

All FT-IR spectra exhibit a broad band centered at ca. 3300 cm^−1^, which can be assigned to the stretching vibrations of O–H groups from water and to the symmetric and asymmetric stretching vibrations of the N–H bonds in silk. Symmetric and asymmetric stretching modes of CH_2_ groups from silk [76] or from terminal TiO–H groups [77] may also contribute to this broad signal.

Bands at 1620 and 1510 cm^−1^ are the amide I and amide II bands of the β-sheet of *B. mori* silk, respectively [78–79]. The band at 1620 cm^−1^ may also be due to the O–H bending vibration from adsorbed water or TiO–H groups [80]. This signal is more intense in the silk/TiO_2_ hybrid materials than in the pure TiO_2_ [81]. Finally, Ti–O–Ti and Ti–O vibration bands are observed between 800 and 400 cm^−1^ region [76]. An identification of the crystal phase (anatase, rutile, brookite) is, however, not possible because of the breadth of these bands.

Figure S7, Supporting Information File 1 shows thermogravimetric analysis (TGA) data. The TGA curves of the pure silk show two steps. The first step which spans up to 105 °C with a 4% weight loss, can be assigned to water desorption. The second step is a large weight loss with three overlapping steps between 210 and 640 °C with an overall 97% weight loss with no further weight loss up to 1000 °C.

Pure TiO_2_ only shows a broad weight loss of 17% from room temperature to 420 °C, which is assigned to water, ethanol, ethylacetoacetate (EtAcAc), and possibly isopropanol from unreacted TiO_2_ precursors [82].

The PEO-containing hybrid materials TPS and TPS_Au_2.5_ essentially exhibit an identical thermal stability. The first weight loss of 9% below 120 °C can be assigned to water and ethanol evaporation. The second step from 120 to 250 °C only shows a minor weight loss of 3% and is likely due to evaporation of more strongly bonded water and ethanol. The third step with an overall weight loss of 20% can again be assigned to silk degradation and combustion of unreacted EtAcAc and TTIP occurring between 250 and 600 °C [82]. No further weight loss is observed up to 1000 °C and the residual 68% can be assigned to TiO_2_.

Like the TPS materials, the TS materials essentially exhibit identical TGA curves irrespective of the presence or absence of Au. Again, the first weight loss of 10% up to 120 °C is due to the loss of water and ethanol. A next weight loss of 3% from 120 to 250 °C is from the evaporation of more strongly bound water and ethanol. The third step is a multi-step silk degradation and combustion of unreacted EtAcAc and TTIP from 250 to 580 °C with an overall weight loss of 28% (TS) and 27% (TS_Au_2.5_) indicating a higher amount of organic matter in the TS materials than in the corresponding TPS materials.

In summary, both types of hybrid material contain of ca. 12% of solvent. The TPS materials contain 20% of organic matter (combined silk and PEO) and the TS materials contain ca. 28% of silk. Consequently, the fraction of TiO_2_ in TPS is ca. 8% higher than in TS.

**Figure 7:**
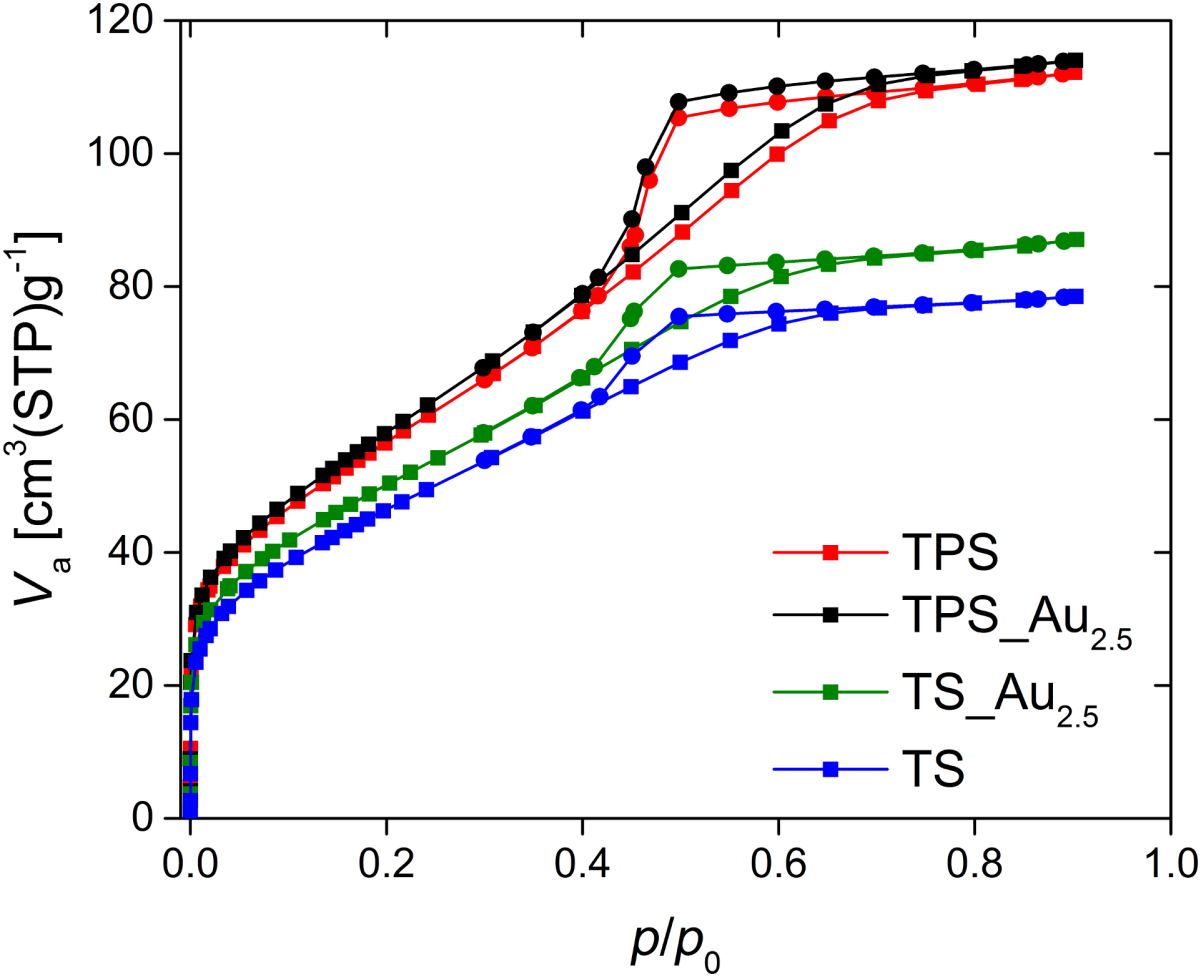
Nitrogen sorption isotherms of TPS (black symbols), TPS_Au_2.5_ (blue symbols), TS_Au_2.5_ (green symbols), and TS (red symbols); ▪ adsorption branch, ● desorption branch.

Figure 7 shows nitrogen sorption data obtained from the Au-free and Au-modified samples. All samples show a type IV(a) isotherm with a type H2(a) hysteresis loop; the hysteresis loop is typical for materials with pore diameters wider than 4 nm when measuring at 77 K [83]. The vertical slope in the beginning of all measurements indicates the presence of micropores and the remainder of the adsorption and desorption branches indicates the presence of mesopores.

Pure TiO_2_ made by the same synthesis method, but without PEO and silk, has a pore diameter of 2.4 nm and a specific surface area of 280 m^2^/g. The TPS and TPS_Au_2.5_ samples have the same pore diameter of 3.2 nm and nearly the same specific surface area of 210 m^2^/g. The materials made without PEO (TS and TS_Au_2.5_) have a lower pore diameter of 2.4 nm and a lower specific surface area of 170 m^2^/g (TS) and 180 m^2^/g (TS_Au_2.5_) respectively. Table 1 summarizes the surface areas and pore volumes.

**Table 1:** Porosimetry data of the hybrid materials and a TiO_2_ control sample made via the same method but without silk and PEO. Surface areas and pore sizes were calculated via the Barrett–Joyner–Halenda (BJH) or Brunauer–Emmett–Teller (BET) method [84–85].

	BJH		BET	Hg intrusion		
sample	Average pore diameter [nm]	Pore volume [mm^3^/g]	Total specific surface area [m^2^/g]	Average pore diameter [nm]	Pore volume [mm^3^/g]	Total pore surface area [m^2^/g]

TiO_2_	2.4	170	280	–	–	–
TPS powder	3.2	150	210	3.5–5 100–50,000	643	49.5
TPS monolith	–	–	–	3.5–10 100–10,000	332	47.8
TPS_Au_2.5_	3.2	150	210	–	–	–
TS powder	2.4	90	170	500–30,000	517	16.8
TS monolith	–	–	–	3.5–10 500–5,000	344	30.3
TS_Au_2.5_	2.4	110	180	–	–	–

**Figure 8:**
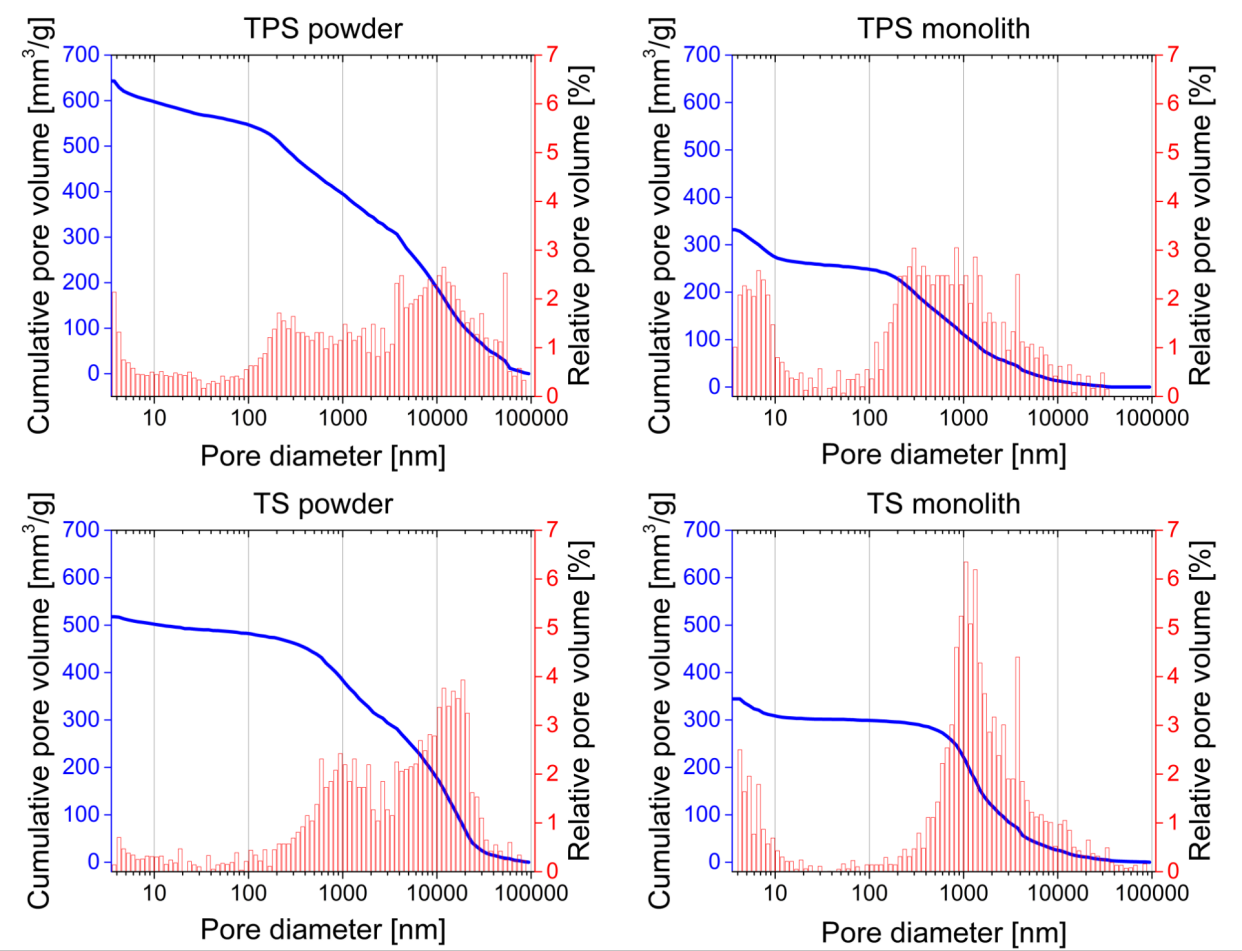
Cumulative (blue) and relative (red) pore volume of TPS (top) and TS (bottom) measured by Hg intrusion.

As SEM and TEM also show the presence of macropores, nitrogen sorption was complemented with Hg intrusion porosimetry. Figure 8 shows that TPS ground to a powder prior to the measurements has a very broad pore diameter distribution. The cumulative pore volume indicates a very undefined porosity with pore sizes mainly in the range of 200 nm and 50 µm. In contrast, in the case of monolithic TPS samples (i.e., samples that were not ground prior to analysis), the pore size distribution is different. Pores created between powder particles by grinding (pores bigger than 4–6 µm) cannot be detected here. As a result, the residual porosity appears more distinct, showing pores with a diameter below 10 nm and in the range of 200 nm and ca. 5 µm.

Both TS samples (ground and monolithic) have a narrower pore size distribution than the TPS samples. In the powdered sample, the pores are mainly on the order of 500 nm to 30 µm whereas the monolithic sample shows pores below 10 nm and from 500 nm to 5 µm with a rather pronounced main pore diameter at 1 µm.

As the purpose of the materials presented here is photocatalytic water splitting, the bandgap is important. It was determined via solid state UV–vis reflection spectroscopy via Kubelka–Munk analysis [61]. The indirect bandgap for all materials is 3.15 ± 0.10 eV, regardless of the presence or absence of Au. This band gap is slightly lower than values found in the literature, which are typically around 3.2 eV for anatase (which is the major component in the current materials) [31,86].

**Figure 9:**
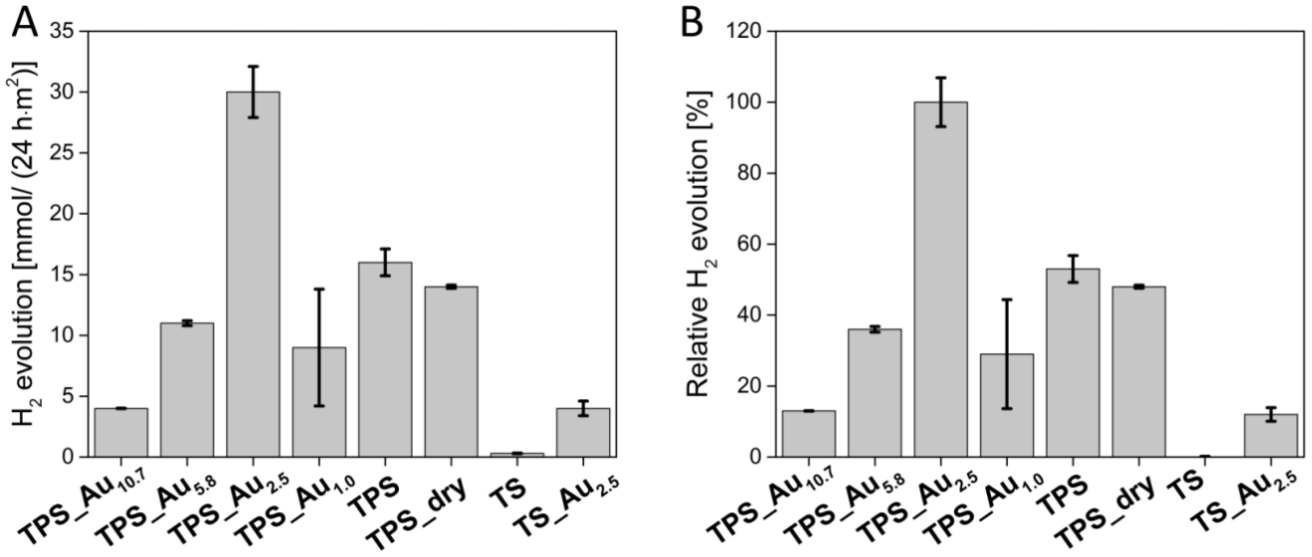
H_2_ production of the hybrid materials using a sunlight simulator. A) Numbers are mmol of H_2_ produced per 24 h and m^2^ of catalyst B) relative H_2_ evolution in %.

Figure 9 shows the results from photocatalytic water splitting experiments in the presence of ethanol as sacrificial reagent. A possible mechanism of photocatalytic water splitting is shown in Equations S1–S5, Supporting Information File 1. The data reveal a significant influence of the amount of Au present in the samples on the photocatalytic efficiencies. TPS_Au_10.7_ shows the lowest H_2_ production of 4 mmol in 24 h (all values are normalized to a catalyst area of 1 m^2^). TPS_Au_5.8_ produces 11 mmol of H_2_, TPS_Au_1.0_ generates 9 mmol, and TPS_Au_0_ produces 16 mmol of H_2_. The highest amount (30 mmol) of H_2_ is produced with TPS_Au_2.5_.

Replacement of the sun simulator with a 300 W Xenon lamp increases the amount of H_2_ produced from TPS_Au_5.8_ by a factor of about 18 from 11 mmol to 200 mmol (Figure S8, Supporting Information File 1). Interestingly, there is no significant difference between materials that were dried before use and those used directly without drying; 16 mmol vs 14 mmol in 24 h.

The TS materials were used as synthesized and show a low H_2_ production of 0.3 mmol in 24 h whereas Au modified TS_Au_2.5_ produces 4 mmol in 24 h.

## Discussion

H_2_ is among the most attractive fuels for a more resource-efficient energy management. As a result, practical and cheap production and delivery strategies for H_2_ gas are highly sought after [6–10]. Photocatalytic water splitting is among the most promising technologies for point-of-use H_2_ production and therefore a large number of photocatalysts have been developed [8,87–90]. As pointed out in the introduction, however, NP-based photocatalysts have disadvantages, for example in terms of recycling.

The current report therefore focuses on a new hybrid material for water splitting. The main advantage of the current group of materials is that they are available as macroscopic, rather uniform, discs (Figure 1). The macroscopic dimensions enable simple handling of the catalysts. A further advantage that needs to be explored in more detail in the future is the fact that the scaffold is made from silkworm silk, which is – at least in principle – a renewable raw material.

The materials are macroscopically quite uniform. Electron microscopy (Figure 3) and porosimetry (Figure 8) show that all materials exhibit a complex pore architecture. The latter can be tuned by the addition of PEO to the reaction mixture. The specific surface area of TiO_2_ without silk and PEO is 280 m^2^/g, which is close to the theoretical external surface (315 m^2^/g) of 5 nm TNP and consistent with earlier data on 6 nm TNP with a surface area of 250 m^2^/g [91].

Generally, the TPS and TS samples have a somewhat lower surface area of 210 m^2^/g and 170 m^2^/g, respectively. These data clearly show that the approach is flexible and enables the synthesis of materials with a tunable pore size. This may be helpful for optimizing the photocatalytic activity.

HRTEM, FFT, and XRD (Figure S4, Supporting Information File 1 and Figure 4) demonstrate that in all cases anatase with traces of brookite forms and that AuNPs are present in the samples. Furthermore, the chemical composition was ascertained via EA, EDXS, XPS, TGA, as well as IR and Raman spectroscopy (Table S4, Supporting Information File 1, Figure 2, Table S3, Supporting Information File 1, Figure S7, Supporting Information File 1, Figure 6) and the data consistently show that the TS samples contain slightly more organic (i.e., silk) material. The deviations in the chemical composition that are observed between the XPS and the EA data are likely due to the fact that EA probes the entire material while XPS solely probes the surface. Indeed XPS sputter profiles (Figure S6, Supporting Information File 1) reveal a carbon-rich surface of the TPS sample suggesting that the surface may be enriched in silk and/or PEO.

Overall, these data prove that: (i) The materials have an open pore structure, (ii) the AuNP and the TNP are in close contact with each other, (iii) the TNPs and the AuNPs are crystalline, and (iv) the materials vary somewhat depending on the synthesis protocols. In spite of this, all materials share the same generic features such as an open porosity and chemical composition suitable for water splitting.

Indeed, all materials are photocatalysts for water splitting. However, Figure 9 clearly shows that there are significant differences between the materials. Expectedly, water splitting is much more efficient under UV irradiation than under simulated sunlight. This is due to the bandgap of 3.15 eV. Indeed it has been pointed out that larger TNP could lead to a higher photocatalytic activity [92] but this aspect is beyond the scope of the current article.

Besides TNP size, the AuNPs play a crucial role. In the current study, samples made with 2.5 mg of HAuCl_4_·3H_2_O yield the best results. Higher Au concentrations possibly lead to an AuNP layer, which is too dense for efficient energy transfer between AuNPs and TNPs. On the other hand, a lower amount of HAuCl_4_·3H_2_O could lead to smaller particles and consequently to very few reaction centers or the reduction of the Au plasmon in the materials, thereby reducing the overall effectiveness of the photocatalytic activation of the reaction.

Comparison of the efficiency of the current materials with other materials is difficult: (i) most photocatalysts described in the literature [22–24] are used as nanoparticles in dispersion. This results in a much higher surface area than those observed in the TS or TPS materials; (ii) the amounts of H_2_ produced are often given in µmol/h or µmol/g catalyst. While this is generally viable, solid materials such as the TS and TPS materials are much better characterized by H_2_ production in µmol/(h·m^2^) as the irradiated area of catalyst is the crucial parameter (and not the mass of catalyst); (iii) there are no standard procedures to quantify the photocatalytic activity and experimental setups show large variations in terms of, e.g., reactor design, feed design, light source, irradiation window, and water/organic solvent mixtures in the reactors [9].

Regardless of this, Silva et al. [24] reported that a higher amount of AuNPs results in a lower H_2_ yield, similar to the data presented here. Chen et al. [23] demonstrated that a high amount of AuNP leads to AuNP clusters, which promote the recombination of electron–hole-pairs. The same studies also show an effect of the irradiation wavelength.

Matsuoka et al. [31] deposited TiO_2_ on a quartz glass plate. The TiO_2_ (columnar rutile) had a height of 1.2 µm and was further sputter-coated with Pt particles. The resulting composites produced 2760 mmol/(24 h·m^2^) of H_2_ with a 500 W Xe lamp. This is higher than the current materials, which produce 200 mmol/(24 h·m^2^) of H_2_. It must be noted, however, that these lower numbers were achieved using a 300 W Xe lamp. Moreover, the material used for these experiments (TPS_Au_5.8_) has only ca. 36% of the efficiency of the best material (TPS_Au_2.5_). In addition, Matsuoka et al. used a rutile-based material with a rutile bandgap of ca. 3.02 eV, whereas the current materials are mainly anatase with a bandgap of 3.15 eV; presumably this slightly larger band gap results is a somewhat less effective use of the irradiated light compared to the example by Matsuoka.

As a result, there are differences between these materials making a quantitative comparison between the different approaches difficult. Nevertheless, TiO_2_-based composites are highly promising and the current example further broadens the pool of attractive and tunable photocatalysts for water splitting. The performance of the current materials is comparable to other photocatalysts, but they have the additional advantage that they can easily be purified and recycled by simple washing.

## Conclusion

We have successfully synthesized two new TiO_2_-silk hybrid materials using *B. mori* silk as a renewable and versatile scaffold. The hybrid materials have a complex architecture and are efficient water splitting catalysts. The TPS-based materials are better catalysts than the TS-based materials but more information is necessary to better understand and to optimize these systems. Although the current efficiency of the catalysts is reasonable, the materials presented here are among the first models of complex water splitting catalysts that are at least partly based on renewable materials that can easily be recycled by simple washing, and are rather efficient at generating H_2_, one of the most attractive green energy sources available today.

## Supporting Information

File 1 Additional data. A comparison of the different hybrid materials, and further analytic results like (STEM, HRTEM, digital microscope images, XRD, binding energies from XPS measurements and results from TGA and elemental analysis) are shown in the supporting information. Furthermore a comparison between H_2_ productions using simulated sunlight vs a xenon lamp and a possible mechanism for photocatalytic water splitting is also shown.
