# Supplementary material for: Bombyx mori silk/titania/gold hybrid materials for photocatalytic water splitting: combining renewable raw materials with clean fuels
Source: Beilstein J Nanotechnol. 2018 Jan 17;9:187–204. doi: 10.3762/bjnano.9.21 (PMC5789386; doi:10.3762/bjnano.9.21)
Supplement: File 1 — Additional data. A comparison of the different hybrid materials, and further analytic results like (STEM, HRTEM, digital microscope images, XRD, binding energies from XPS measurements and results from TGA and elemental analysis) are shown in the supporting information. Furthermore a comparison between H2 productions using simulated sunlight vs a xenon lamp and a possible mechanism for photocatalytic water splitting is also shown. [file Beilstein_J_Nanotechnol-09-187-s001.pdf]

Supporting Information
for

Bombyx mori silk/titania/gold hybrid materials for photocatalytic water splitting: combining renewable raw materials with clean fuels

Stefanie Krüger[1], Michael Schwarze[2], Otto Baumann[3], Christina Günter[4], Michael Bruns[5], Christian Kübel[6], Dorothée Vinga Szabó[5], Rafael Meinusch[7], Verónica de Zea Bermudez[8] and Andreas Taubert*[1,§]

Address: [1]Institute of Chemistry, University of Potsdam, D-14476 Potsdam, Germany, [2]Institute of Chemistry, Technical University Berlin, D-10623 Berlin, Germany, [3]Institute of Biochemistry and Biology, University of Potsdam, D-14476 Potsdam, Germany, [4]Institute of Earth and Environmental Science, University of Potsdam, D-14476 Potsdam, Germany, [5]Institute for Applied Materials (IAM) and Karlsruhe Nano Micro Facility (KNMF), Karlsruhe Institute of Technology (KIT), D-76344 Eggenstein-Leopoldshafen, Germany, [6]Institute of Nanotechnology (INT) and Karlsruhe Nano Micro Facility (KNMF), Karlsruhe Institute of Technology (KIT), D-76344 Eggenstein-Leopoldshafen, Germany, [7]Institute of Physical Chemistry, Justus-Liebig-University Giessen, D-35392 Giessen, Germany and [8]Department of Chemistry and CQ-VR, University of Trás-os-Montes e Alto Douro, Pt-5001-801 Vila Real, Portugal

Email: Andreas Taubert - ataubert@uni-potsdam.de

* Corresponding author

[§] Tel.: +49 (0) 331 977 5773

Additional data

Table S1: Hybrid materials studied in this work. Note that only the main components are given. For details of sample preparation and composition see experimental section below.

	TS	TS_Au$_x$	TPS	TPS_Au$_x$
m (PEO_{780}) (mg)<br><br>m (PEO_{8300}) (mg)	-<br><br>-	-<br><br>-	200<br><br>200	200<br><br>200
m (Silk in silk solution) (mg)	800	800	400	400
V ($Ti(OiPr)_4$) (mL)	5.0	5.0	5.0	5.0
m ($HAuCl_4 \cdot 3H_2O$) (mg)	-	2.5<br><br>5.6<br><br>6.1	-	1.0<br><br>2.5<br><br>5.0<br><br>10.7

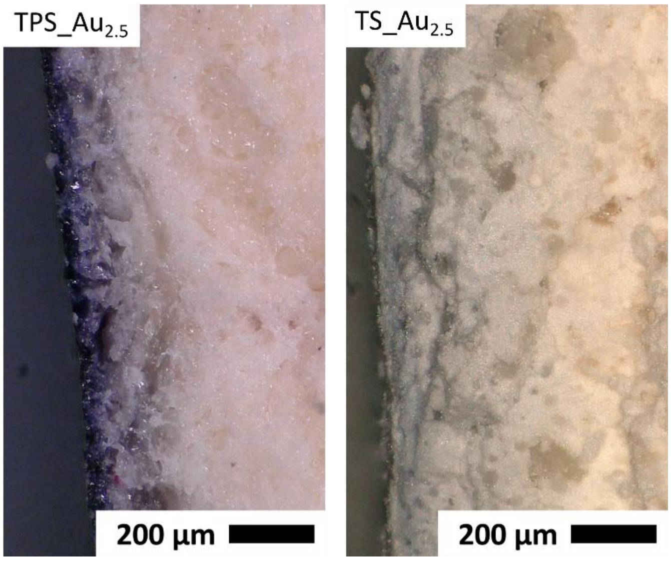

Figure S1: Digital microscopy images of TPS_Au$_{2.5}$ and TS_Au$_{2.5}$. Top surface of the material is on the left hand side of both images.

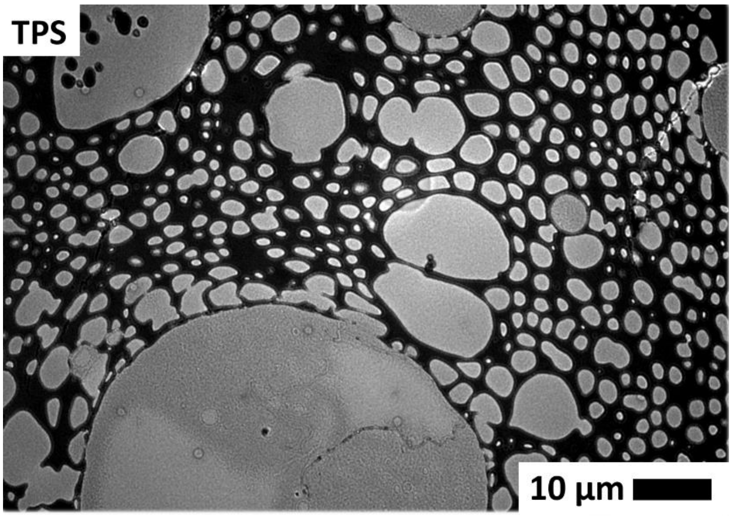

Figure S2: TEM image of TPS with a larger pore of around 50 µm (bottom of image).

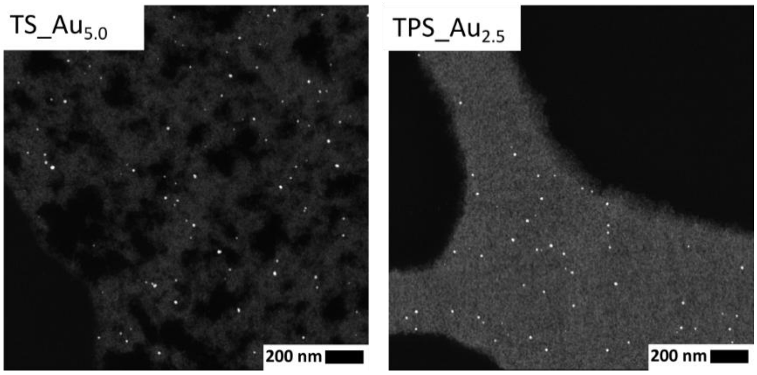

Figure S3: Dark field STEM (DF-STEM) image of TS_$Au_{5.0}$ and TPS_$Au_{2.5}$. The light spots are the AuNP, the gray background is the titania/silk hybrid and black areas are holes or regions with very little material.

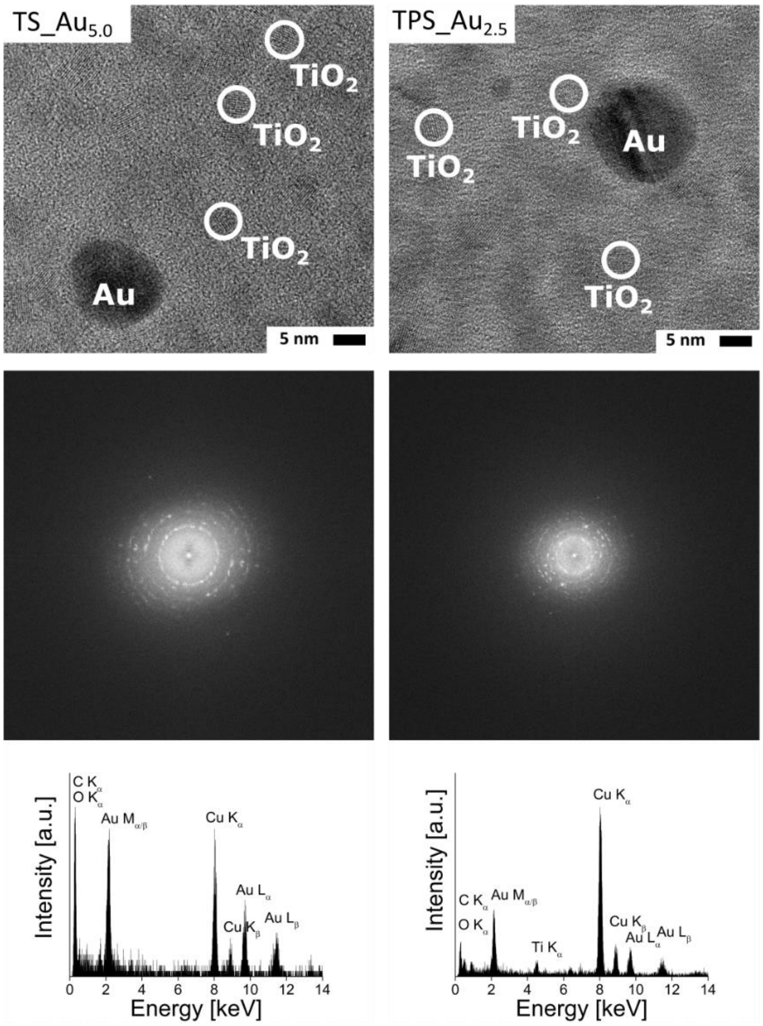

Figure S4: HRTEM images of TS_$Au_{5.0}$ and TPS_$Au_{2.5}$ along with the corresponding FFT images and EDX spectra. The copper signals stem from stray signals from the Cu-TEM grid. Carbon and oxygen signals may originate from TiO_2, the surrounding silk, and/or embedding resin.

Table S2: Lattice spacings and corresponding hkl index assignments of the FFT evaluated using PASAD [1].

measured TS_Au$_{5.0}$		anatase [ICDD 98-015-4602]		brookite [ICDD 00-029-1360]		Au [ICDD 00-004-0784]	
**K [1/nm]**	**d [nm]**	**hkl**	**d [nm]**	**hkl**	**d [nm]**	**hkl**	**d [nm]**
2.821	0.354	011	0.353	120	0.351		
3.501	0.286			121	0.290		
4.231	0.236	004	0.237	131	0.237	111	0.236
5.238	0.191	020	0.190	231	0.189		
5.871	0.170	015	0.170	320	0.169		
4.179[a]	0.239					111	0.236
4.937[a]	0.203					200	0.204
**measured TPS_Au$_{2.5}$**		**anatase [ICDD 98-015-4602]**		**brookite [ICDD 00-029-1360]**		**Au [ICDD 00-004-0784]**	
**K [1/nm]**	**d [nm]**	**hkl**	**d [nm]**	**hkl**	**d [nm]**	**hkl**	**d [nm]**
2.868	0.349	011	0.353	120	0.351		
4.693	0.213			221	0.213		
5.162	0.194	020	0.190	032	0.197		
5.930	0.169	015	0.170	320	0.169		
4.166[a]	0.240					111	0.236
4.937[a]	0.203					200	0.204

[a]Reflections measured directly on the particle.

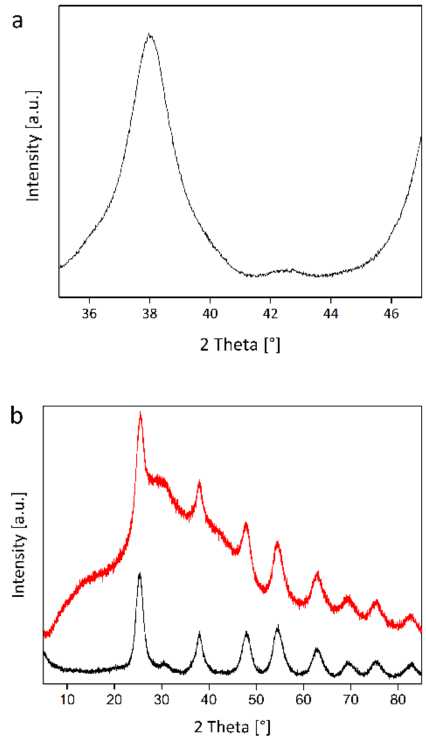

Figure S5: a) XRD pattern of TPS_$Au_{5.0}$ highlighting the region between 35-47°. b) XRD patterns of a wet (red line) and dry (black line) TPS hybrid material.

Table S3: XPS binding energies, assignments to the respective binding partners, and atomic concentrations for the samples TS_Au$_{2.5}$ and TPS_Au$_{2.5}$.

		TPS_Au$_{2.5}$ [atom %]	TS_Au$_{2.5}$ [atom %]	
Peak	Binding energy [eV][a]	Atomic Concentration [%]	Atomic Concentration [%]	Assignment
Au $4f_{7/2}$	84.0	1.2	2.2	Au^0 [2]
C 1s	285.0	16.2	23.9	C-C/ C-H [3-6]
	286.4	21.0	20.2	C-N/ C-O [3-7]
	288.3	16.7	16.4	O=C-N/ O=C-OH [3-5,7]
N 1s	400.1	16.8	16.4	C-N-H/ O=C-N [6-9]
	402.0	0.5	0.6	$C-N^+H_x$ [3]
Ti $2p_{3/2}$	459.0	2.1	0.2	TiO_2 [10]<br>Ti-O-C [11]
O 1s	530.5	4.7	-	TiO_2 [10]
	531.8	16.9	17.0	O=C-N/ O=C [3,6]<br>Ti-OH [12]
	533.3	3.5	3.2	C-O [3,6]<br>Ti-OH [12]

[a]The binding energies are referenced to the C 1s = 285.0 eV energy of hydrocarbons. In consequence the uncertainty for weak peaks (even for Au $4f_{7/2}$) is ±0.2 eV.

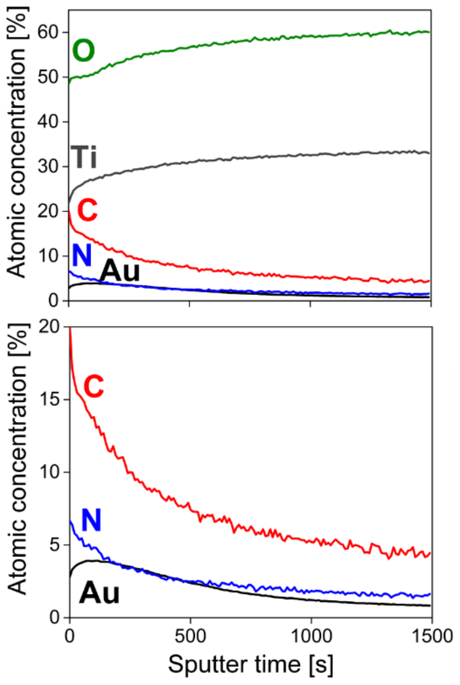

Figure S6: XPS sputter depth profiles of the $TPS\_Au_{2.5}$ surface (top) and a zoom in (bottom). Note: as it was not possible to determine the sample thickness [13], the data are represented vs. sputter time.

Table S4: Chemical composition of the hybrid materials obtained from EA.

sample	C ± 0.3 %	H ± 0.3 %	N ± 0.3 %	total amount CHN
TiO_2 [a]	1.5	1.2	0	2.7
silk	46.2	7.2	17.9	71.3
TPS	9.5	2.4	3.4	15.2
$TPS\_Au_{2.5}$	9.1	2.3	3.3	14.6
TS	13.3	2.9	4.9	21.2
$TS\_Au_{2.5}$	13.2	2.8	4.9	20.8

[a] The CHN amount in TiO_2 comes from unreacted $Ti(OiPr)_4$ and EtAcAc.

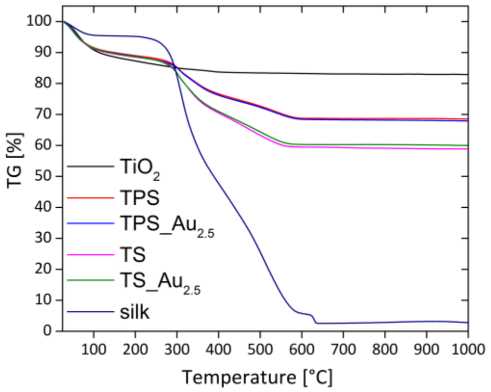

Figure S7: TGA curves of TiO_2 (black line) TPS (red line), TPS_Au$_{2.5}$ (light blue line), TS (violet line), TS_Au$_{2.5}$ (green line) and *B. mori* silk (dark blue line).

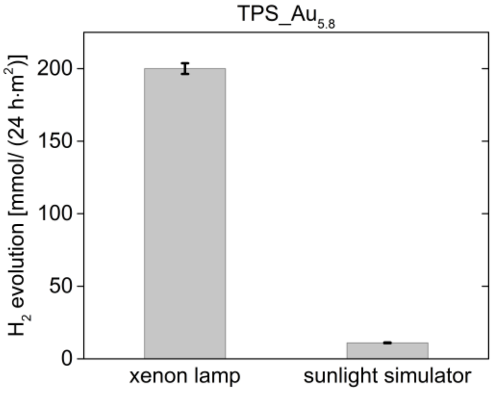

Figure S8: H_2 production of TPS_Au$_{5.8}$ using a sunlight simulator or a xenon lamp.

A possible mechanism for water splitting in the presence of ethanol in shown in Equation S1–S5 (adapted by Chen et al. [14])

$TiO_2 + h\nu \rightarrow h^+ + e^-$ (S1)

$H_2O + h^+ \rightarrow \cdot OH + H^+$ (S2)

$\cdot OH + CH_3CH_2OH \rightarrow CH_3\cdot CHOH + H_2O$ (S3)

$CH_3\cdot CHOH + \cdot OH \rightarrow CH_3CH(OH)_2 \rightarrow CH_3CHO + H_2O$ (S4)

$2\ H^+ + 2\ e^- \rightarrow H_2$ (S5)

References

1 C. Gammer, C. Mangler, C. Rentenberger and H. P. Karnthaler, *Scr. Mater.*, 2010, **63**, 312–315.

2 Y. Joseph, I. Besnard, M. Rosenberger, B. Guse, H. Nothofer, J. M. Wessels, U. Wild, A. Knop-

Gericke, D. Su, R. Schlögl, A. Yasuda and T. Vossmeyer, *J. Phys. Chem. B*, 2003, **107**, 7406–7413.

3 N. Zydziak, C. M. Preuss, V. Winkler, M. Bruns, C. Hübner and C. Barner-Kowollik, *Macromol. Rapid Commun.*, 2013, **34**, 672–680.

4 E. H. Lock, D. Y. Petrovykh, P. Mack, T. Carney, R. G. White, S. G. Walton and R. F. Fernsler, *Langmuir*, 2010, **26**, 8857–8868.

5 C. Rodriguez-Emmenegger, C. M. Preuss, B. Yameen, O. Pop-Georgievski, M. Bachmann, J. O. Mueller, M. Bruns, A. S. Goldmann, M. Bastmeyer and C. Barner-Kowollik, *Adv. Mater.*, 2013, **25**, 6123–6127.

6 S. Engin, V. Trouillet, C. M. Franz, A. Welle, M. Bruns and D. Wedlich, *Langmuir*, 2010, **26**, 6097–6101.

7 C. M. Preuss, T. Tischer, C. Rodriguez-Emmenegger, M. M. Zieger, M. Bruns, A. S. Goldmann and C. Barner-Kowollik, *J. Mater. Chem. B*, 2014, **2**, 36–40.

8 M. Bruns, C. Barth, P. Brüner, S. Engin, T. Grehl, C. Howell, P. Koelsch, P. MacK, P. Nagel, V. Trouillet, D. Wedlich and R. G. White, *Surf. Interface Anal.*, 2012, **44**, 909–913.

9 T. Tischer, A. S. Goldmann, K. Linkert, V. Trouillet, H. G. Börner and C. Barner-Kowollik, *Adv. Funct. Mater.*, 2012, **22**, 3853–3864.

10 I. Tunc, M. Bruns, H. Gliemann, M. Grunze and P. Koelsch, *Surf. Interface Anal.*, 2010, **42**, 835–841.

11 B. Qiu, Y. Zhou, Y. Ma, X. Yang, W. Sheng, M. Xing and J. Zhang, *Sci. Rep.*, 2015, **5**, 8591.

12 J. Yan, G. Wu, N. Guan, L. Li, Z. Li and X. Cao, *Phys. Chem. Chem. Phys.*, 2013, **15**, 10978–88.

13 Mai, T.; Wolski, K.; Puciul-Malinowska, A.; Kopyshev, A.; Gräf, R.; Bruns, M.; Zapotoczny, S.; Taubert, A. Anionic Polymer Brushes for Biomimetic Calcium Phosphate Mineralization – a Surface with Application Potential in Biomaterials. *Biomacromolecules*, in press.

14 X. Chen, S. Shen, L. Guo and S. S. Mao, *Chem. Rev.*, 2010, **110**, 6503–6570.
